# Comparing the performance of machine learning methods in estimating the shear wave transit time in one of the reservoirs in southwest of Iran

**DOI:** 10.1038/s41598-024-55535-2

**Published:** 2024-02-27

**Authors:** MohammadRasool Dehghani, Shahryar Jahani, Ali Ranjbar

**Affiliations:** Faculty of Petroleum, Gas and Petrolchemical Engineering, Petroleum Engineering Department, Persian Gulf University, Bushehr, Iran

**Keywords:** Shear wave transit time, Shear wave velocity, Geomechanics, Machine learning, Crude oil, Solid Earth sciences

## Abstract

Shear wave transit time is a crucial parameter in petroleum engineering and geomechanical modeling with significant implications for reservoir performance and rock behavior prediction. Without accurate shear wave velocity information, geomechanical models are unable to fully characterize reservoir rock behavior, impacting operations such as hydraulic fracturing, production planning, and well stimulation. While traditional direct measurement methods are accurate but resource-intensive, indirect methods utilizing seismic and petrophysical data, as well as artificial intelligence algorithms, offer viable alternatives for shear wave velocity estimation. Machine learning algorithms have been proposed to predict shear wave velocity. However, until now, a comprehensive comparison has not been made on the common methods of machine learning that had an acceptable performance in previous researches. This research focuses on the prediction of shear wave transit time using prevalent machine learning techniques, along with a comparative analysis of these methods. To predict this parameter, various input features have been employed: compressional wave transit time, density, porosity, depth, Caliper log, and Gamma-ray log. Among the employed methods, the random forest approach demonstrated the most favorable performance, yielding R-squared and RMSE values of 0.9495 and 9.4567, respectively. Furthermore, the artificial neural network, LSBoost, Bayesian, multivariate regression, and support vector machine techniques achieved R-squared values of 0.878, 0.8583, 0.8471, 0.847 and 0.7975, RMSE values of 22.4068, 27.8158, 28.0138, 28.0240 and 37.5822, respectively. Estimation analysis confirmed the statistical reliability of the Random Forest model. The formulated strategies offer a promising framework applicable to shear wave velocity estimation in carbonate reservoirs.

## Introduction

Shear wave velocity is one of the important parameters in petroleum engineering and geomechanical modeling. The importance of shear wave velocity in petroleum engineering is significant in many ways^1,2^. Without accurate information about the shear wave velocity in an oil reservoir, geomechanical modeling cannot fully describe and predict the rock behavior in that reservoir^3^. In the field of geomechanical modeling, having high accuracy and precision in shear wave velocity estimation provides important information about rock properties, rock mechanical behavior, and pressure transfer in hydrocarbon reservoirs^4^. By using geomechanical modeling, it is possible to analyze and predict rock behavior in different reservoir conditions and oil and gas-related issues according to shear wave velocity and other relevant parameters, including rock density and resistance. For example, geomechanical modeling can be used in the analysis of reservoir pressure, rock fractures, crumpling of drill pipes, surface subsidence, and reservoir compaction, optimal design of well stimulation operations such as hydraulic fracturing, as well as simulation of rock behavior in porous environments^5,6^. As a result, accurate estimation of shear wave velocity is very important in geomechanical modeling and enables petroleum engineers to gain a complete understanding of rock properties and reservoir performance. This information is useful in choosing appropriate reservoir behavior scenarios, production planning, and increasing reservoir productivity^7^.

Shear wave velocity, as one of the significant properties of rock, indicates its hardness and resistance, and its accurate understanding provides valuable information about rock's mechanical properties, plasticity, and rock behavior in response to mechanical actions^8^. In addition to using this parameter in geomechanical modeling, shear wave velocity also provides effective data about fractures, cracks, and rock weaknesses in oil reservoirs. By analyzing the shear wave velocity around fractures and rock weak points, they can be detected and their impact on reservoir behavior and performance can be modeled^2^. This information can be used in decisions related to hydraulic fracturing, acid injection, or creating appropriate mechanical stresses. In the following, some aspects of the importance of shear wave velocity are introduced^9,10^:

- Determination of mechanical properties of rock: shear wave velocity is directly related to the mechanical properties of rock, including hardness, and strength. By having the most accurate possible estimate of shear wave velocity, we can significantly improve the determination of these properties and make the best decisions about drilling, injecting, and extracting oil.
- Diagnosis and analysis of fractures: shear wave velocity can help us identify fractures and weak points in rock. By analyzing the shear wave velocity around fractures, we can obtain important information about the location, size, and properties of fractures. This information is invaluable for evaluating their behavior, optimizing operations, and managing the reservoir.
- Relationship with other parameters: shear wave velocity in combination with compressive velocity provides more complete information about oil reservoirs. By having detailed information about these parameters, we can improve the reservoir pressure estimation, detect the mechanical behavior of the reservoir rock, estimate the mechanical stresses, and improve the productivity of the reservoir.
- Basic data in geomechanical modeling: as stated, the shear wave velocity is one of the most basic data in the construction of mechanical earth models, whose applications were mentioned above.

Shear wave velocity can be measured through laboratory or field methods. In the laboratory method, the core sample taken from the well is stimulated directly using shear waves, and when the shear waves reach a point in the rock, the time of their passage is recorded. According to the distance between the excitation source and the detector (identifier/receiver), shear wave velocity is calculated. It is noteworthy that this method, which is known as the direct method, despite its high accuracy, requires a lot of time and money, as well as expert manpower. Also, coring and conducting direct tests to estimate the physical and mechanical properties of the rock is expensive and difficult in many cases due to its destructive nature. Alternatively, indirect methods such as using seismic data or petrophysical data taken from well logs can be used with acceptable accuracy. This is possible by using statistical analysis of well logging and seismic data as well as using empirical relationships extracted by researchers. Also, today, this can be done with high accuracy using artificial intelligence algorithms^11^.

The estimation of shear wave velocity using empirical relations has been introduced by many researchers. Among these, we can mention the relationships presented by^12–16^. In these models, different empirical relationships have been proposed to estimate V_s_ using only pressure wave velocity (V_P_) in different rocks. One of the most important disadvantages of experimental methods is the lack of use of other petrophysical parameters such as density, porosity, etc., which have an effective relationship with shear wave velocity. Also, since these methods are generally developed for specific lithology or field and geological conditions, they are not comprehensive and cannot be generalized to different types of fields with different lithological conditions. Along with experimental methods, machine learning systems and intelligent algorithms can be very effective and used comprehensively. In the following, some prominent studies conducted in this field are mentioned.

Tabari et al.^17^ used artificial neural networks to estimate shear wave velocity in underground formations. Data obtained from various well log measurements such as pressure wave velocity, gamma, neutron, and density have been used in this study. The neural network was trained using 80% of the data and validated with the remaining 20%. This study showed that using V_P_ is more accurate and reliable than using porosity to approximate shear wave velocity. The mentioned study highlights the potential of machine learning algorithms for the accurate estimation of subsurface parameters such as shear wave velocity using borehole data^17^.

In 2015, Norafken and Kodkhodaei used machine learning algorithms such as NF, GA, ANN, and ACOFIS intending to improve shear wave velocity estimation. The well logging data of pressure wave velocity, density, and neutron porosity were used as input to the four mentioned algorithms and the performance of different algorithms was investigated. The results showed that the new and combined approach mentioned in that study (ACOFIS model), in addition to the successful estimation of the shear wave velocity, is also able to estimate other reservoir parameters. In the mentioned study, the ACOFIS model showed the lowest error and the highest correlation coefficient, so that the Vs values predicted using the ACOFIS model were in good agreement with the measured values^18^.

In 2019, Bokar et al. conducted a study to increase the accuracy of predicting shear wave velocity values through machine learning algorithms using well log data. They used conventional well logging data such as caliper, gamma, neutron, density, shear and compression wave transit time, resistivity, water saturation, and total porosity. Algorithms used in this study include various regression methods and support vector machines. The results showed that the Gaussian Exponential Process regression model has the lowest root mean square error and shows the best model. The findings of this study demonstrate a notable enhancement in the precision of prognostications as compared to the linear regression model and furthermore underscore the potential of the approach utilized in reservoir intervals. The results of this study also accentuate the significance of employing machine learning algorithms to achieve precise assessments of reservoir rock properties^19^.

In 2020, Zhang et al. used the Bayesian method to determine the shear wave velocity in a Chile Formation in South China. This study used data from various well logs, including measurements of gamma rays, pressure wave velocity and shear wave velocity, volume fraction, neutron, density, and saturation components. This study showed that the Bayesian approach is more efficient in estimating the shale formation velocity than the usual methods. The findings of this study can be valuable for reservoir exploration and identification in shale formations^20^.

Olayiwola et al. in 2021 using MD, GR, RHOB, NPHI, RES-SHT, RES-MED, RES-DEP, CAL data, and machine learning algorithms (ANN, ANFIS, LLSVM, regression) on a comprehensive dataset of About 6526 data points from an oil field in the northern Norwegian basin were used to estimate V_s_ and V_p_. Based on the reported results, the LSSVM model is the most accurate technique for estimating both V_p_ and V_s_. The accuracy order of the models was reported as LSSVM > ANFIS > ANN > REG^21^.

Zhang et al. conducted a study in 2022 to develop a model for lithology prediction using well log data. Various types of input data and machine learning algorithms have been used in the mentioned study. CNN model was reported as the most accurate model with a 4.2% improvement compared to other models. The performance ranking of the algorithms used in this study is CNN > SVR > ANN. In this study, 768 training and testing sets with time series features were used^22^.

In 2023, a study was undertaken by Kheirollahi et al. to develop a precise model to predict shear wave velocity. This was accomplished through the utilization of data derived from an oil field located in the northern basin of Norway. In this study, different well logging measurements and machine learning algorithms, including MLR, ELM, and ANN, were used. The dataset contained 455 data points and pre-processing was performed before applying the algorithms. The feed-forward neural network exhibited the utmost level of precision, whilst a profound artificial neural network was posited for the prognostication of the target response in additional wells. The model underwent adjustment via the network search optimization technique to procure the optimal configuration^23^.

In 2023, Rajabi et al. conducted a study to develop an accurate model for predicting V_P_ using logs including GR, RHOB, NPHI, RES-SHT, and average resistance. They used machine learning algorithms such as Melm-PSO, Melm-ga, and CNN. According to the results, the CNN model is the most accurate model for predicting VP, followed by Melm-PSO and Melm-ga^1^.

In 2023, Feng et al. conducted studies to develop an accurate model for predicting shear wave velocity using the deep neural network (DNN) algorithm. The DNN model was very accurate in velocity prediction, with errors of less than 5% in both laboratory and field domains. The findings of this study highlight the potential of using DNN algorithms to estimate subsurface features^24^.

By reviewing the studies, it seems that most of the studies in this field have used machine learning algorithms and limited well logging data, and a comprehensive study that evaluates and compares all widely used machine learning methods to shear wave velocity estimation, It is less noticeable.

In this study, in addition to using various types of well logging data related to a reservoir in the southwest of Iran, common machine learning algorithms also be used to estimate Vs. Therefore, perceptron multilayer artificial neural network, random forest, Bayesian, generalized least squares, multivariate linear regression, and support vector machine methods have been used. These data were taken from one of the wells of a hydrocarbon field in the southwest of Iran in the Darian Formation. This formation is a carbonate formation with dolomite and calcite units. Furthermore, an evaluation has been conducted in each of the methods to determine the impact of the parameters on the estimation of shear wave velocity. The novelty of this research can be compared to the commonly utilized machine learning techniques for the assessment of shear wave velocity in a particular carbonate reservoir located in the southwestern region of Iran. Additionally, the analysis estimates the influence of distinct petrophysical parameters on shear wave velocity assessment within each of the methods. One of the main differences between the present study and previous researches is the application and simultaneous comparison of a common set of machine learning methods in shear wave estimation in a carbonate reservoir. This is seen as a shortcoming and a lack of literature in previous studies.

## Methodology

### Available data and studied reservoir formation

In this study, the petrophysical data set of the Wire-line logs, one of the wells located in the oil field in the southwest of Iran, in the area of Dezful subsidence, has been used. The data set is related to the depth of 4305–4554 from the well in front of the Darian Formation, which is a carbonate formation. In this research, several petrophysical well logs have been used as input to intelligent methods to create a relationship for shear wave velocity estimation. Finally, the finest method using selected statistical parameters and the method that provides the best performance is used to forecast shear wave velocity in other wells in the field that lack shear wave data. The data used include shear wave transit time, compression wave transit time, gamma, depth, neutron, density, and diameter measurement. The statistical information of the data sets and their distribution is presented in Table 1. Knowing the statistical characteristics of the data can help the process of removing outliers and also build a suitable machine learning model for prediction. From statistical data, information such as dispersion, frequent data, average data, average deviation, etc. can be obtained.

**Table 1:** Statistical information of the data used in the study.

	DTS	DTC	GR	HCAL	HTNP	RHOZ
Mean	116.3657	62.65156	25.21375	6.192439	0.102329	2.4623
Std	13.72256	9.913601	9.198272	0.801655	0.062093	0.110064
Median	118.1413	62.75445	22.9317	5.863	0.1074	2.449
Mode	105.0251	65.52693	22.2379	5.7711	0.0185	2.3791
Kurtosis	− 1.23967	− 0.46232	2.041928	13.04231	− 1.1655	− 0.52548
Skewness	− 0.02531	− 0.05874	1.361315	3.002278	− 0.0282	0.289958
Min	88.82855	38.99416	9.0195	5.6562	0.0029	2.1002
Max	145.3697	86.66428	65.0151	12.1149	0.2337	2.7615

**Figure 1:**
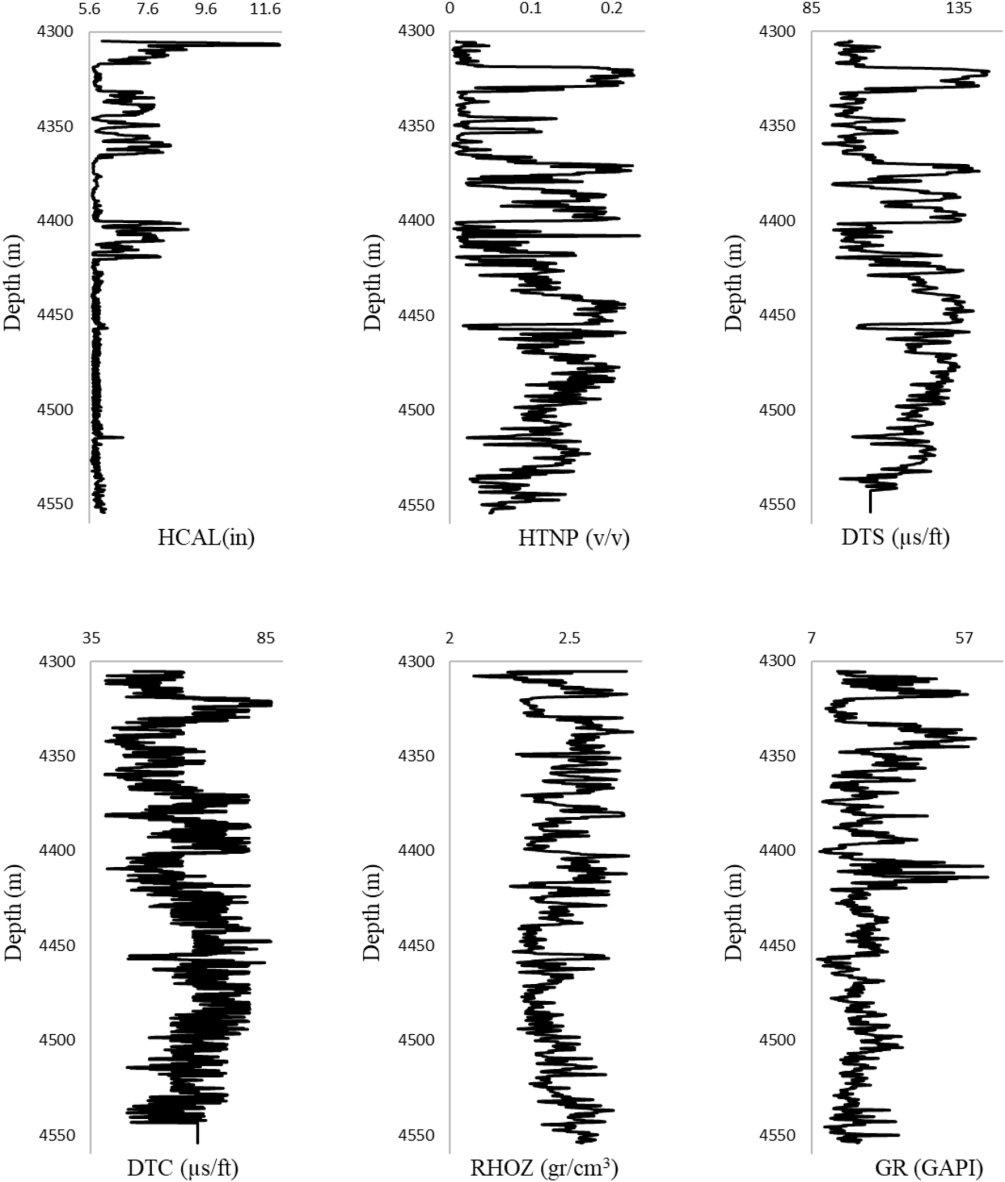
Profiles of the log parameters used versus depth.

The standard deviation represents the dispersion of the data, DTS, DTC, GR, HCAL, RHOZ, and HTNP data have the highest dispersion, respectively. Positive kurtosis means that the data has a greater deviation from the mean, as a result, GR and HCAL data have a greater deviation from the mean compared to other parameters. Also, the skewness values indicate the accumulation of data on the sides of the average. If the value of skewness is positive, it means that the data tends more to the left of the mean, and if it is negative, it means that the data tends more to the right of the mean. GR, HCAL, and RHOZ data are to the left of the mean and DTS, DTC, and HTNP data are to the right of the mean. Figure 1 shows the profile of input data relative to depth.

**Figure 2:**
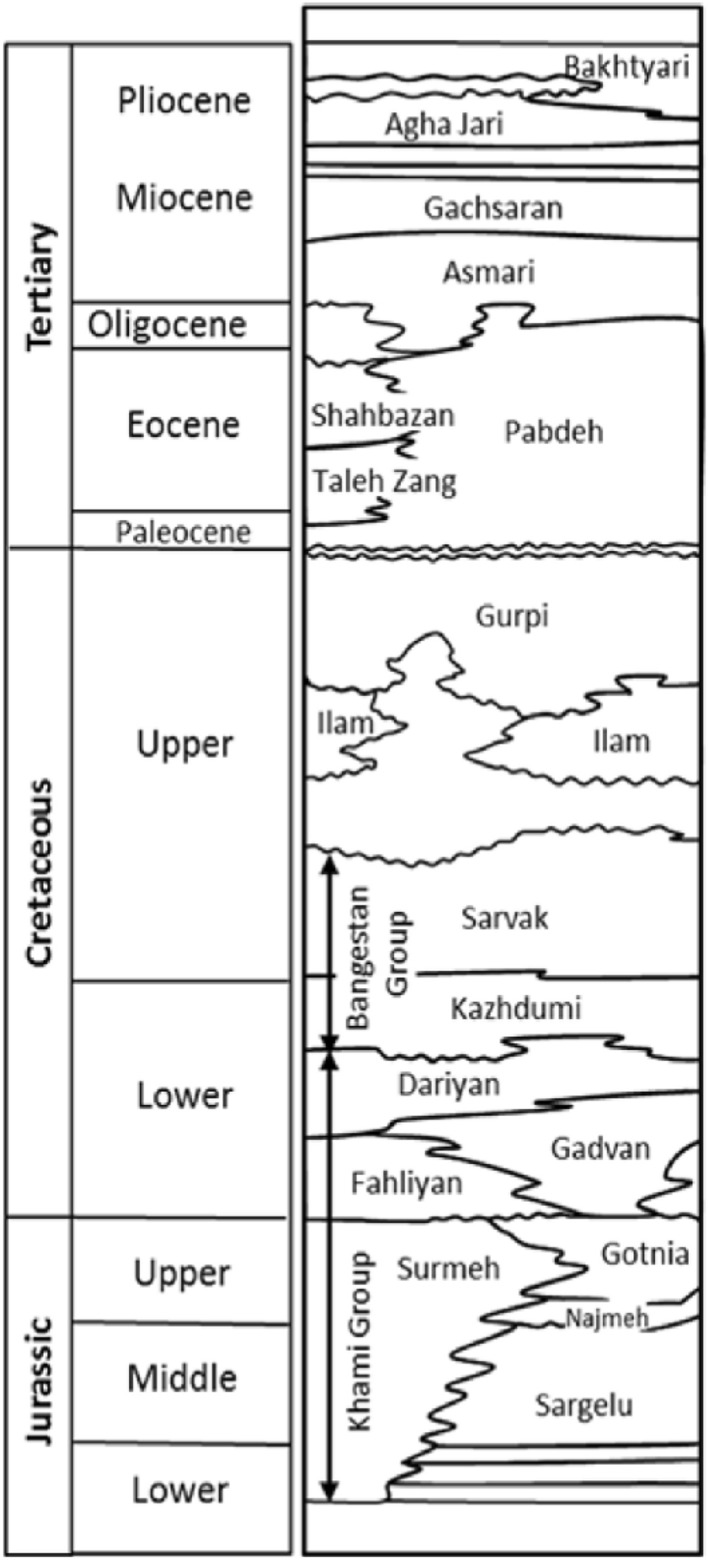
Stratigraphic column of Dezful subsidence formations (Zeinalzadeh 2020).

As mentioned, the studied reservoir formation is the Darian Formation. Darian Formation is one of the recognizable geological formations in the southwest of Iran. This formation is located in parts of Khuzestan, Kohgiluyeh and Boyer Ahmad, Lorestan and Chaharmahal and Bakhtiari provinces. Darian Formation usually includes limestone, shale, and clay sedimentary masses with variable thicknesses and due to its wide coverage, it can show different characteristics in different areas. This formation is considered a hydrocarbon reservoir. The age of the Darian Formation in Dezful depression is reported as the Lower Cretaceous. This formation is located in the Khami group. Its upper border is formed by Kazhdami carbonates and its lower border is formed by Fahlian formations and in some areas by Gadvan formations. The stratigraphic column of Dezful subsidence formations and the placement of the Darian Formation are shown in Fig. 2.

### Data preprocessing and outlier data removal

In data mining, removing outliers is very important and is considered an important step in data preprocessing. Outlier data refers to data that differs significantly from the general patterns of other data and unexpectedly compromises some of the quality and accuracy of the data. Removing outlier data can have several effects^25^. Below are some of the importance of removing outlier data^26–28^

- Improving modeling accuracy: outlier data may cause irrational deviations in the models and make the estimates inaccurate.
- Reducing the impact of noise: by removing outlier data, the effect of noise and undesirable deviations in data analysis can be reduced.
- Improving the quality of data and interpretation of phenomena: by removing outliers, we can focus more on important data patterns and relationships.

To pre-process the data and remove outliers, spacing methods such as Z-score, modified Z-score, standard deviation, Tukey, adjusted boxplot, median, and median absolute deviation have been chosen by researchers^29^. Besides the mentioned methods, graphic methods can also be used.

**Figure 3:**
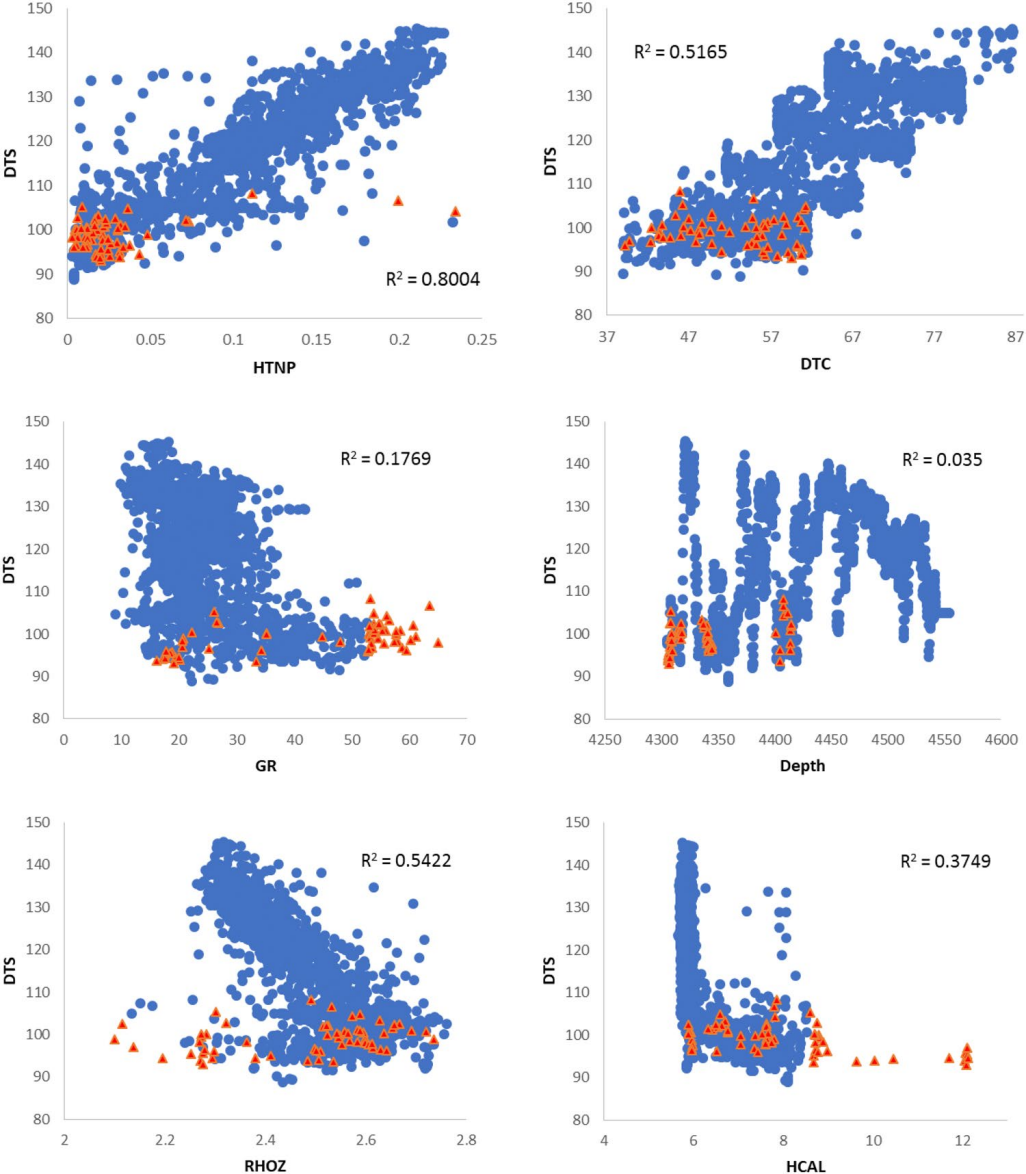
Correlation diagram of data used with shear wave velocity and outlier data display.

In this research, outlier data were removed using the standard deviation method. The standard deviation method is a commonly used statistical technique that involves calculating the standard deviation of a dataset and then removing any data points that fall outside a certain number of standard deviations from the mean. This method uses two powerful estimators, which are defined as follows^29^:

1 $$2SD\, Method: \overline{x }\pm 2SD$$

2 $$3SD\, Method: \overline{x }\pm 3SD$$

where $\overline{x }$ is the mean and SD is the standard deviation. Data that do not fall within these ranges are known as outlier data^30^. This method is applied to symmetric data that follow a normal distribution and is a powerful method for large normal data^31^. Figure 3 shows the correlation of the input data as well as the deleted outlier data.

In Fig. 3, the red points represent the deleted data from the input data series. It should be noted that by removing an outlier from a parameter, the entire row of data including other parameters is removed. That is why the red points in some graphs are exactly between the blue points.

### Machine learning methods

#### Neural network method

**Figure 4:**
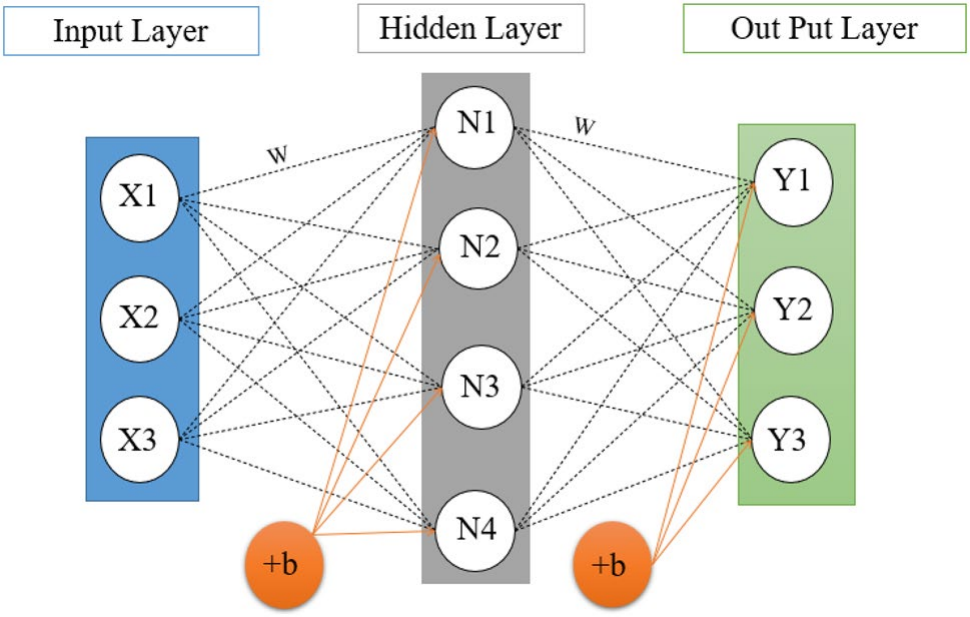
Multilayer artificial neural network.

The function of neural networks is similar to that of biological neural systems because they both consist of a group of interconnected neurons. Important components of a neural network include the input layer, output layer, weights, bias, and activation function^32,33^. The input layer, which forms the first layer of the network, is responsible for receiving raw information. The performance of the hidden layers is determined according to the inputs and the relationship between the weights and biases. The performance of the output layer depends on the hidden layers and the weight. Weights determine the influence of each feature of the input on the network and the bias of the influence of each input data^34^. The difference between the output of the artificial neural network and the target data is called the error function. Equation (3) shows the relationship between input bias and weights^32^.

3 $$Net=\sum {{\text{W}}}_{{\text{ij}}} {{\text{X}}}_{{\text{i}}}+{b}_{j}$$

where W_ij_ represents the weights, X_i_ the input data and b_j_ the bias. By choosing the optimal weight and bias, a logical relationship between input and output can be established. The optimality of the weight and bias values is very important in the performance of the system, that's why the mean square error method is used for the optimal selection of these parameters. The data generated by the initial hidden layer undergoes transfer function processing to nullify the neurons that exert minimal influence on the outcome or lead to system deviation. Equation (4) is one of the most important transfer functions, the sigmoid function^35^ which is written as follows:

4 $${\text{F}}\left(Net\right)=\frac{1}{1+{{\text{e}}}^{-{\text{Net}}}}$$

where F(Net) represents the activity values of each neuron and Net represents the neuron output of each layer. Figure 4 shows a schematic of the multilayer artificial neural network.

#### Bayesian algorithm

The Bayesian algorithm is one of the supervised learning methods in artificial intelligence. The history of the Bayesian algorithm dates back to the year 1763 when the Bayes formula was discovered by Thomas Bayes, but the equation of this algorithm was created in 1980^36,37^. The performance of this algorithm is based on probabilistic and statistical methods. The Bayesian method is based on the Bayes rule, which allows to determination of probabilistic estimates for model parameters using previous information and new data^38^.

In Bayesian regression, only the probability distribution of the parameters is used instead of a fixed value for them. In other words, instead of finding an optimal value for the parameters, the probability distribution of the parameters is obtained. This probability distribution shows the probability that each parameter has a certain value. By having a probability distribution of parameters, we can calculate predictions and estimates that take uncertainty into account^39^.

One of the common methods in Bayesian regression is the use of prior distribution and posterior distribution. The prior distribution shows how much probability we give each value initially, and the posterior distribution shows how much probability we give each value after seeing the data. For inference and prediction in Bayesian regression, the posterior distribution is used. To calculate the posterior distribution, we first calculate the analytical or numerical posterior distribution using the prior distribution and the observed data. Then, using the posterior distribution, we can calculate estimates such as the mean, variance, confidence interval, and predictive distribution^40,41^.

In Bayesian regression, the governing equations of this method include Bayes' theorem and the Markov chain rule. In general, the Bayes equation and the rule of the Markov chain can be expressed as follows^42,43^:

5 $$P\left(\left.\theta \right|D\right)= \frac{P\left(\left.D\right|\theta \right)P\left(\theta \right)}{P\left(D\right)}$$

where $P\left(\left.\theta \right|D\right)$ is the posterior distribution of the parameters (θ) considering the observed data (D). $P\left(\left.\theta \right|D\right)$ Expresses the similarity function (Likelihood function) and shows how much data corresponds to the conditions that the parameters (θ) have. P(θ) is the prior distribution of the parameters, which shows how likely we believe different values for the parameters before seeing the data. P(D) is the normalization constant, whose inverse ratio makes the posterior distribution conform to the principles of probability.

##### Markov chain rule

6 $$P\left(\left.{\theta }_{1},{\theta }_{2},\dots ,{\theta }_{n}\right|D\right)=P\left({\theta }_{1}\right)\cdot P\left(\left.{\theta }_{2}\right|{\theta }_{1}\right)\cdot P\left(\left.{\theta }_{3}\right|{\theta }_{1},{\theta }_{2}\right)\cdot \dots \cdot P\left(\left.{\theta }_{n}\right|{\theta }_{1},{\theta }_{2},\dots ,{\theta }_{n-1}\right)\cdot P\left(\left.D\right|{\theta }_{1},{\theta }_{2},\dots ,{\theta }_{n}\right)$$

where ${\theta }_{1},{\theta }_{2},\dots ,{\theta }_{n}$ are the parameters of the regression model and $P\left(\left.{\theta }_{i}\right|{\theta }_{1},{\theta }_{2},\dots ,{\theta }_{i-1}\right)$ is the prior distribution of the parameter θ_i_ considering the parameters ${\theta }_{1},{\theta }_{2},\dots ,{\theta }_{i-1}$ is Also, $P\left(\left.D\right|{\theta }_{1},{\theta }_{2},\dots ,{\theta }_{n}\right)$ is the function of similarity/accuracy of data considering all parameters^42^.

In the Bayesian method, our goal is actually to find the posterior distribution of ${\text{P}}\left(\left.\uptheta \right|{\text{D}}\right)$, which gives us more information about the parameters and their uncertainty.

#### Random forest method

Random Forest algorithm is a powerful machine learning method that is based on the combination of several decision trees. Random forest is used in regression and estimation problems and is very popular due to its generalizability, robustness to interactions, and good performance on new data. In the random forest algorithm, several decision trees are generated. Each decision tree is made separately and based on the division of input variables and their values, as a decision regression. This means that each leaf of the decision tree estimates the regression value that is extracted based on the training data for that leaf^44^.

A unique feature of random forest is that each decision tree is constructed using a random subset of input features. In other words, instead of using all the features to build each tree, several random features are chosen and the tree is built based on them. This stochastic process for feature selection leads to an increase in diversity and resolution between trees and allows the random forest model to respond to the data with high accuracy and good fit.

After building each decision tree, regression values are predicted for new samples. Finally, the final regression value for each sample is calculated as the average of the regression values of all decision trees. In this way, random forest can provide accurate predictions for regression and estimation problems.

Among the advantages of the random forest algorithm, the following can be mentioned^44,45^:

- Interpretability: according to the structure of the decision tree, it is easy to understand which features are important for prediction.
- Resistance to noisy data: random forest has a good ability to deal with noisy data and shows good performance for new data.
- Eliminating interactions: by using several decision trees, random forest can effectively reduce the interactions in the data.
- High speed: due to the distribution of calculations between trees, random forest can be implemented in parallel and has a high speed in general.

It should be noted that the random forest algorithm may tend to overfitting in some cases. Methods such as choosing the right number of trees and limiting the depth of trees can be used to deal with this problem. The random forest algorithm in regression is characterized by two equations: one for constructing decision trees and another for calculating the ultimate output of the random forest^46^.

##### The decision tree construction equation

In each step of decision tree construction, input variables are randomly selected and based on these variables, the tree is constructed. In general, the decision tree construction equation is as follows:

$$ \begin{gathered} {\text{X}}_{{\text{k}}} = {\text{RandomSubset}}\left( {{\text{X}},{\text{m}}} \right) \hfill \\ {\text{SplitVariable}} = {\text{SelectBestSplitVariable }}\left( {{\text{X}}_{{\text{k}}} } \right) \hfill \\ {\text{SplitValue}} = {\text{SelectBestSplitValue}}\left( {{\text{SplitVariable}},{\text{X}}_{{\text{k}}} } \right) \hfill \\ {\text{Xleft}} = \{ {\text{x}} \in {\text{X}}_{{\text{k}}} |{\text{x}}\left[ {{\text{SplitVariable}}} \right] \le {\text{SplitValue}}\} \hfill \\ {\text{Xright}} = \{ {\text{x}} \in {\text{X}}_{{\text{k}}} |{\text{x}}\left[ {{\text{SplitVariable}}} \right] > {\text{SplitValue}}\} \hfill \\ {\text{LeftChild}} = {\text{BuildTree}}\left( {{\text{X}}_{{{\text{left}}}} } \right) \hfill \\ {\text{RightChild}} = {\text{BuildTree}}\left( {{\text{X}}_{{{\text{right}}}} } \right) \hfill \\ {\text{Tree}} = \left( {{\text{SplitVariable}},{\text{SplitValue}},{\text{LeftChild}},{\text{RightChild}}} \right) \hfill \\ \end{gathered} $$

In this equation, X represents the training data set and m denotes the number of features that are selected each time the decision tree is built^46^.

##### The equation for calculating the final output of the random forest

**Figure 5:**
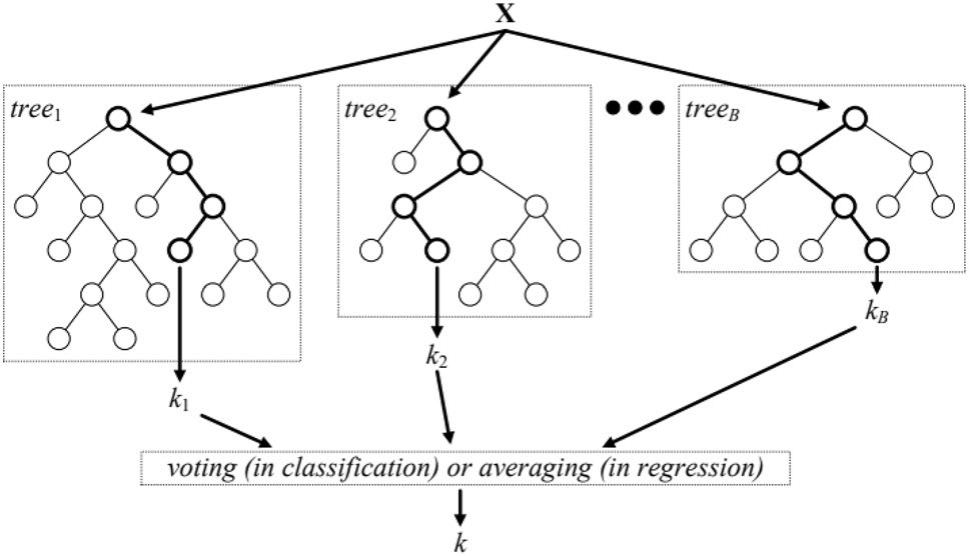
Schematic of random forest algorithm^47^.

After building a certain number of decision trees, the final output of the random forest is calculated for an input sample. The equation used to calculate the final output in regression is as stated below:

7 $$\widehat{Y}= \frac{1}{T}{\sum }_{i=1}^{T}{Prediction}_{i}(X)$$

where $\widehat{Y}$ represents the estimated output for input $X$, $T$ denotes the total number of trees random forest, and ${Prediction}_{i}(X)$ represents the output computed by decision tree $i$. Figure 5 shows the schematic of the random forest method.

#### Least-squares boosting method

Regression tree ensembles are models used for predictions, generated by combining different regression trees, each with its weight. LSBoost is a regression suite designed to minimize the mean squared error. The steps of LSBoost method are as follows:

1. Base model: first, a base model (for example, a linear support vector machine) is defined, which is considered the first model in the set of models.
2. Prediction values: using the base model, prediction values are calculated for the training samples.
3. Calculation of model error: the calculation of model error involves determining the difference between the predicted values and the actual values. In regression tasks, the typical approach to measuring this error is by utilizing the sum of squared errors.
4. Building a new model: a new model is built based on the calculated errors. In LSBoost, this new model is built to reduce the error. Usually, the support vector machine is used with the kernel function.
5. Combination of models: the new model is combined with the previous models so that the final model is used for estimation. This combination is usually done using a weighting factor that is based on the calculated errors.
6. Repeating steps 2–5: steps 2–5 are repeated until reaching the best model for estimation. Usually, the number of models (number of steps) is determined by the user.

By repeating the above steps, LSBoost tries to build a stronger and more advanced model for estimating regression values by combining simple models. This method is widely used due to its power and effectiveness in estimation and regression^48^.

The governing equations of the LSBoost method for estimation and regression are as follows:

8 $${\widehat{f}}_{0}\left(t\right)={\text{argmin}}{\sum }_{j=1}^{n}{({y}_{j}-\gamma )}^{2}$$

Here ${\widehat{f}}_{0}\left(t\right)$ represents the base model, which is considered the first model in the set of models. To estimate the target value of y, a constant value of $\gamma$ is chosen so that the sum of squared errors (SSE) is minimized.

1. Building a new model:
  9 $${\widehat{f}}_{m}\left(t\right)={\widehat{f}}_{m-1}\left(t\right)+ \lambda \cdot {h}_{m}(t)$$
  Here ${\widehat{f}}_{m}\left(t\right)$ denotes the boosted model that is built in step m of LSBoost. This model is combined with the sum of the previous model ${\widehat{f}}_{m-1}\left(t\right)$ and the new model ${h}_{m}(t)$ which is called the weak learner function. $\lambda$ is a weighting factor that controls how much the new model adds to the previous model.
2. Weak learner
  10 $${h}_{m}\left(t\right)= \underset{h}{\mathrm{arg min}}{\sum }_{i=1}^{n}({{y}_{i}-{\widehat{f}}_{m-1}\left({t}_{i}\right)-h({t}_{i}))}^{2}$$
  Here, ${h}_{m}\left(t\right)$ represents the weakness function constructed at step m. The optimization of this function involves utilizing the previous model, ${\widehat{f}}_{m-1}\left(t\right)$, and aiming to minimize the discrepancies between the actual values of y and the predicted values obtained from the previous model, namely ${\widehat{f}}_{m-1}\left({t}_{i}\right)$^49^.

#### Multivariate regression method

Regression plays a crucial role as a data analysis tool, allowing for the examination of the relationship between independent and dependent variables. Specifically, multivariate regression focuses on identifying the most favorable relationship between several independent variables and the dependent variable^50^. The formula for the prediction function in linear regression is stated as follows:

11 $$h\left(x\right)={\beta }_{0}+{\beta }_{1}{x}_{1}+\dots +{\beta }_{n}{x}_{n}$$

In this context, the input parameter is represented by xi, and the weight coefficient is denoted as ${\beta }_{i}$. The determination of the optimal weight coefficients involves minimizing the objective function. Typically, the objective function is computed by summing the squared errors, allowing for the identification of the best weight coefficients^2^:

12 $$J=\frac{1}{2}\sum_{i=1}^{m}{\left(h\left({x}^{i}\right)-{y}^{i}\right)}^{2}$$

Here, ${x}^{n}$ and ${y}^{n}$ are independent and dependent variables in training samples, respectively, and m is the number of training samples. The main goal in regression is to find the best values for the weight coefficients that will reduce the sum of the squared errors and obtain a more accurate prediction function for the dependent variable.

One of the most important algorithms used in regression and optimization problems is the gradient descent algorithm. The main goal of this algorithm is to minimize an objective function (such as a regression error function). This model has parameters such as regression coefficients, which values should be adjusted in such a way that the objective function (usually the sum of squared errors) is minimized.

The gradient descent algorithm to optimize this model, using the learning rate and the gradient of the objective function, gradually moves the value of the parameters to the minimum value. This algorithm is based on repeated steps as follows:

1. The initial value for the parameters is chosen (usually randomly).
2. The gradient of the objective function for each parameter is computed.
3. The parameters are updated as follows:
  $$\mathrm{Latest parameter value}=\mathrm{previous parameter value}-(\mathrm{learning rate }\times \mathrm{ gradient})$$
4. Steps 2 and 3 are repeated until a specified stopping condition (e.g., a specified number of iterations or reaching a minimum acceptable value) is met.

The learning rate is the rate that determines how much the gradient descent algorithm should move towards reducing the parameter values at each step. If the learning rate is large, the algorithm may move towards an unstable minimum value instead of a local minimum, and instead of optimization, adverse rotations are generated. If the learning rate is small, the algorithm may progress as fast as the local minimum, but may not converge to the minimum value in general^2^.

The governing equation of the gradient descent algorithm in regression is as follows^51^:

13 $${\theta }_{new}= {\theta }_{old}-(LearningRate \times \nabla j(\theta )$$

In this equation, ${\theta }_{new}$ indicates the new value of the parameter (regression coefficients). ${\theta }_{old}$ indicates the previous value of the parameter. learning_rate is the learning rate that determines how much move towards reducing the parameter value in each step. $\nabla j(\theta )$ indicates the gradient of the objective function (regression error function) relative to the parameters. In each step of the algorithm, the new value of the parameter ${\theta }_{new}$ is equal to the previous value of the parameter ${\theta }_{old}$ minus the product of the learning and the gradient of the objective function (∇J(θ)) relative to the parameters.

The primary objective of this algorithm is to iteratively perform these steps until it achieves the minimum value of the objective function and optimizes the parameters.

#### Support vector machine

Support vector regression is a machine learning algorithm used for regression and was introduced in 1995 by Vapnik^52^. The objective of this algorithm is to discover a function that can estimate the output using the available database. Within this algorithm, a subset of training samples known as support vectors is specifically taken into account^53^. The primary objective of this algorithm is to determine a linear correlation between input vectors with n dimensions, utilizing the following equation:

14 $$f\left(x\right)={\theta }^{T}x+\beta $$

To this matter, $\theta$ represents the slope while $\beta$ denotes the deviation of the regression line. To determine the values of these two, the following cost function minimization is required:

15 $$R=\frac{1}{2}{\Vert \theta \Vert }^{2}+\frac{C}{t}\sum_{i=1}^{m}{\left|{y}_{i}-f({x}_{i})\right|}_{\varepsilon }$$

In this context, m refers to the count of training samples, C represents the coefficient controlling the boundary, and ε signifies the cost function employed in Vapnik's support vector regression, introduced as follows^52^.

16 $${\left|{y}_{i}-f({x}_{i})\right|}_{\varepsilon }=\left\{\begin{array}{ll}0 &  if\, \left|{y}_{i}-f({x}_{i})\right|\le \varepsilon \\ \left|{y}_{i}-f({x}_{i})\right|-\varepsilon &   otherwise\end{array}\right.$$

By using support vector regression, the output prediction function is estimated with higher accuracy and can be used in many machine learning problems.

In support vector machine regression, kernel functions are employed to convert the input data into a higher-dimensional space. These kernel functions extract new features from the data in a nonlinear manner, aiding in more precise predictions. Various kernel functions, such as linear, polynomial, radial, etc., are available and chosen based on the nature of the data and the specific problem at hand. The process of training and prediction in regression with support vector machine includes the following steps:

- Model training: in this step, training data with correct labels are used to make the SVM model to make predictions. The SVM algorithm tries to find an optimal surface in the feature space that separates the training data points well and maximizes the covariance between the data points and the surface.
- Determination of parameter values: in SVM, there are parameters such as C and ε, whose values affect the performance and efficiency of the model. In general, the parameter C indicates the amount of error allowed in the training data, and the parameter ε specifies how far from that value it will go to the side of the error.
- Prediction: once the model is trained, the test data is utilized to generate predictions for the corresponding values. In support vector machine regression, the model's output comprises the predicted values for the continuous variables.

### Ethical approval

This material is the authors' own original work, which has not been previously published elsewhere. The paper is not currently being considered for publication elsewhere. The paper reflects the authors' own research and analysis in a truthful and complete manner. The paper properly credits the meaningful contributions of co-authors and co-researchers. The results are appropriately placed in the context of prior and existing research. All sources used are properly disclosed (correct citation). Literally copying of text must be indicated as such by using quotation marks and giving proper references. All authors have been personally and actively involved in substantial work leading to the paper, and will take public responsibility for its content.

## Results and discussion

### Determining the ratio of training to test data

**Figure 6:**
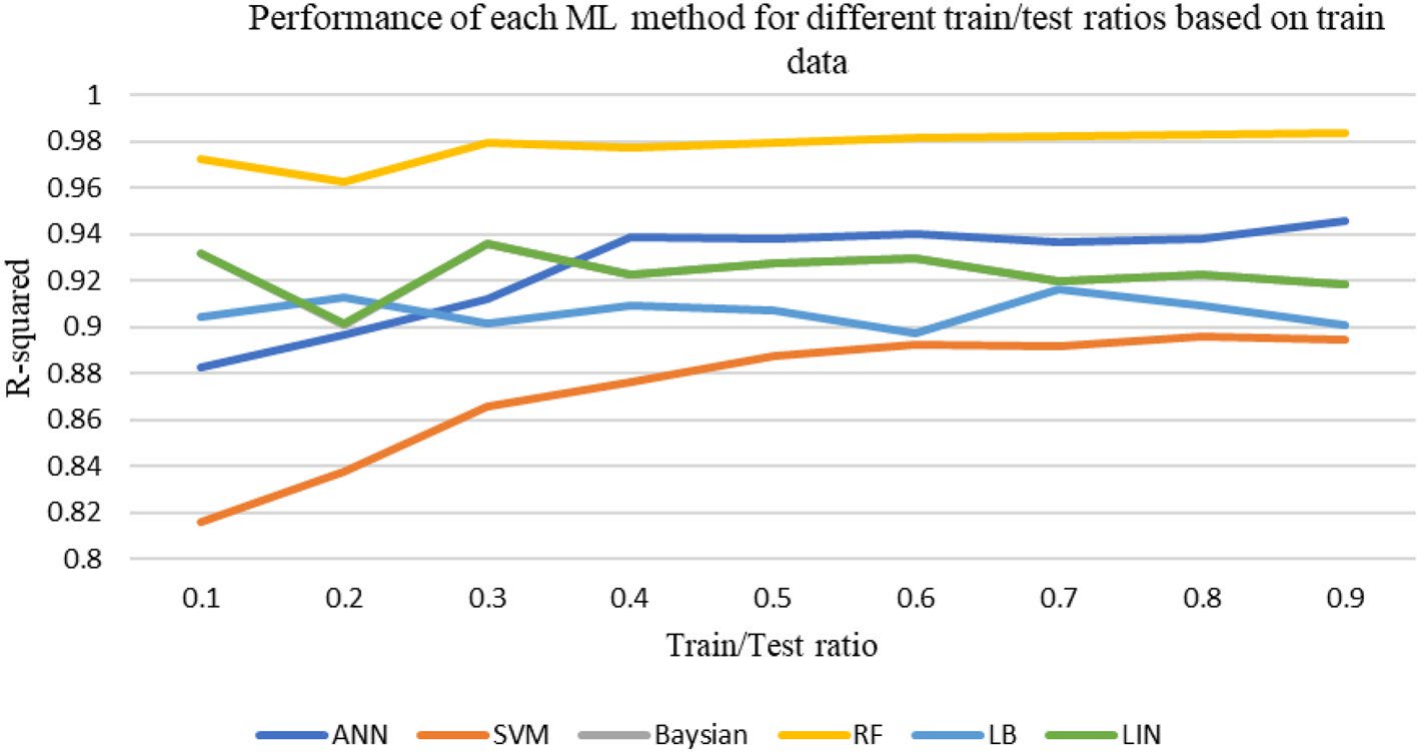
Accuracy of different machine learning methods in different percentages of training-to-test data based on train data.

**Figure 7:**
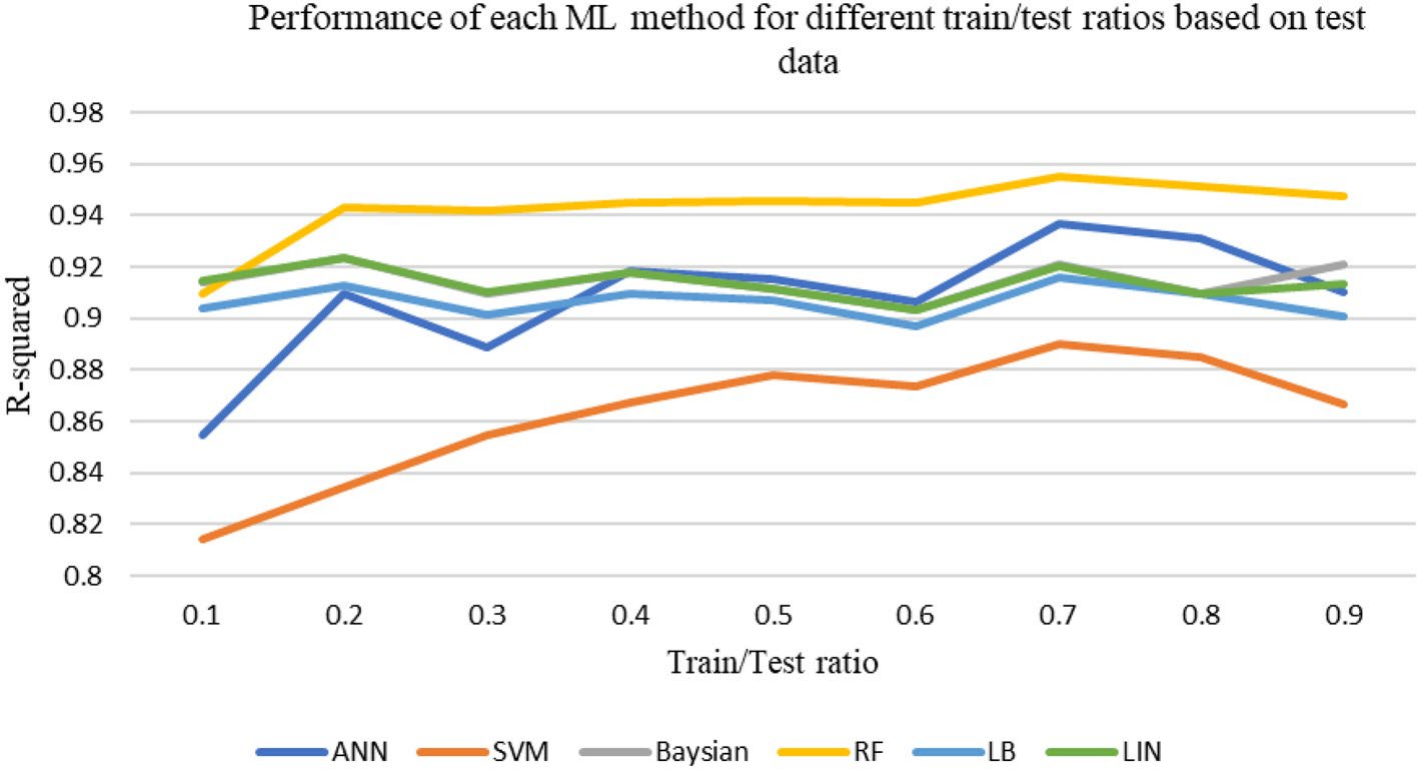
Accuracy of different machine learning methods in different percentages of training-to-test data based on test data.

**Figure 8:**
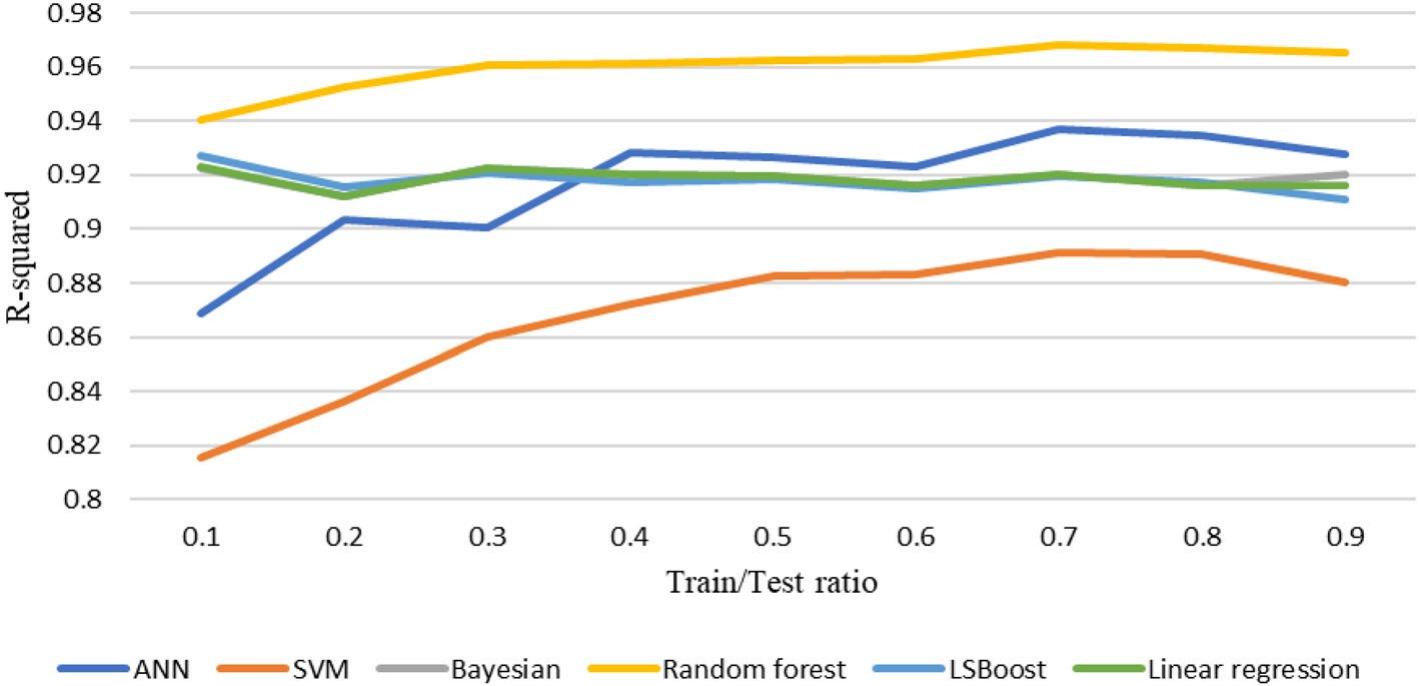
Accuracy of different machine learning methods in different percentages of training-to-test data.

After the initial processing of the data and the removal of outliers, using various machine learning methods described above, the shear wave velocity has been estimated. During this process, the available data is initially divided into two categories: training and testing. In each iteration, a specific portion of the data is chosen for training and testing purposes. The data is randomly selected and fed into the machine learning algorithm code based on the predetermined percentage. After 20 consecutive runs, the correlation (R^2^) and accuracy values of each model are averaged and reported as representative of the overall accuracy of that model. This work has been done for different ratios of data to extract the best ratio. The accuracy of methods at different train-to-test ratios based on test and train data sets are shown in Figs. 6 and 7. Figure 8 shows the accuracy of different methods in different ratios of training to test data. Based on the obtained results, the ratio of 70% generally shows the best accuracy in each method. Therefore, considering the ratio of 70–30 data for training to test, the methods have been compared and the performance of each has been evaluated.

### The performance of machine learning methods in estimating Vs

Comparing the performance of different machine learning methods, including neural networks, Bayesian, linear regression, random forest, LSBoost and support vector machine regression, can be done based on several factors. Below are some of these factors and points of comparison:

- Generalization power: support vector machine regression, Neural networks and random forest, and usually has a high ability to generalize. Due to their powerful structure, they can model more complex patterns and non-linear interactions. On the other hand, linear and Bayesian regression are more suitable for modeling simple patterns and linear interactions.
- Model complexity: neural networks and random forest usually require more complex models and require more parameters. In contrast, linear and Bayesian regression work with simpler models and have fewer parameters. This can directly influence the training time and computational complexity involved.
- Training data: the amount of training data available can also have an impact on the performance of machine learning methods. If the training data is sparse, linear and Bayesian regression may perform better than more sophisticated methods because they suffer less from fitting noisy data. In contrast, if the training data is large and complex, support vector machine regression, neural networks and random forest usually perform better than linear and Bayesian regression.
- Ability to deal with high dimensions: when dealing with high-dimensional data, linear and Bayesian regression encounter challenges due to their limitations in effectively handling a substantial number of features. In this situation, support vector machine regression, neural networks and random forest perform best because they can extract complex features and nonlinear relationships between features.
- Comparability: the scalability of the methods is also very important in their comparison. Support vector machine regression, neural networks and random forest are highly scalable and can adapt to large amounts of data and features. On the other hand, linear regression and Bayesian regression face a large amount of data and considerable computational problems.

**Figure 9:**
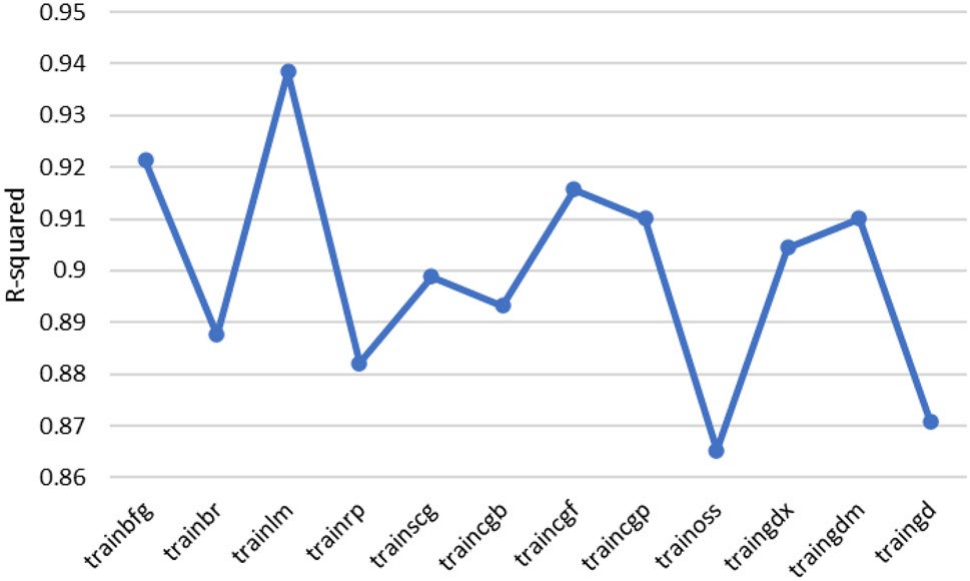
Performance of different training functions in artificial neural network.

**Figure 10:**
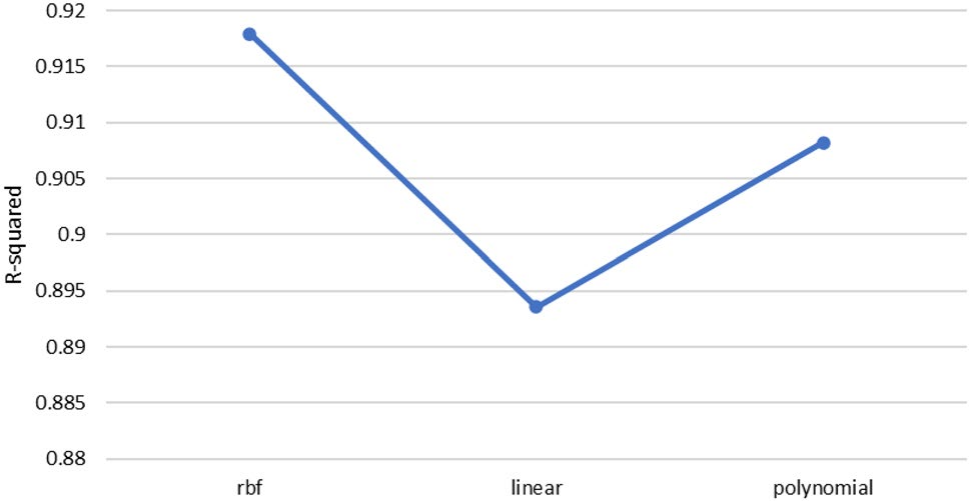
Performance of different kernel functions in SVM.

**Figure 11:**
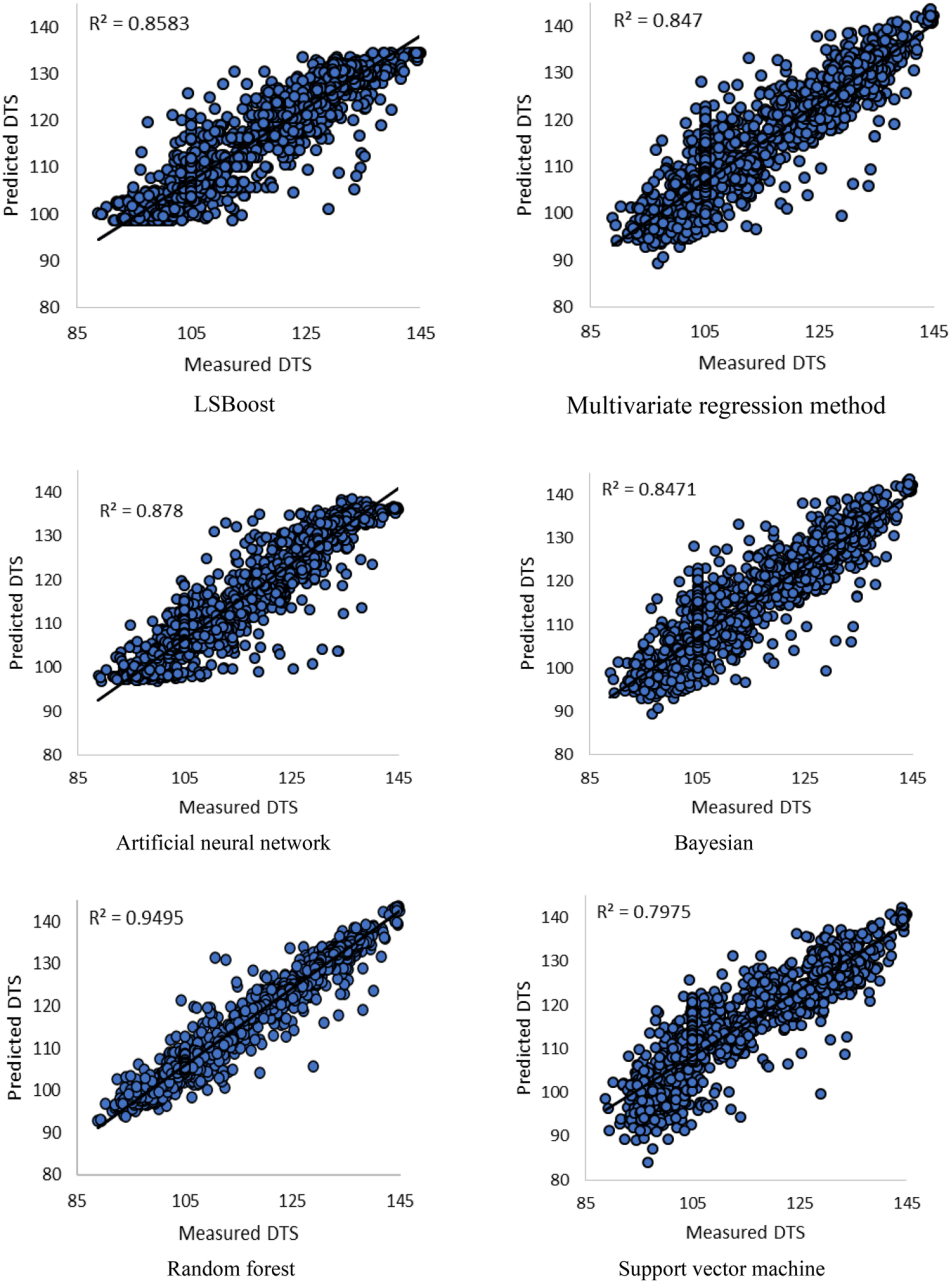
Accuracy of different machine learning methods in estimating Vs.

According to the above explanations, each machine learning method has its advantages and limitations, and choosing the best method depends on the problem in question, available data, and environmental conditions. The performance of each machine learning method employed in this study to estimate shear wave velocity is depicted in Fig. 11. Accordingly, the random forest method provides the best answer with an accuracy of R^2^ = 0.9495. After that, LSBoost, Bayesian and linear regression methods are located with an average value of R^2^ = 0.85. It should be noted that neural network and support vector machine methods showed the lowest accuracy among them. Meanwhile, in the process of training these algorithms, several meta-parameters were used to adjust these methods. In this regard, for neural network method, different types of network architecture, number of layers, number of neurons, types of activation functions such as logistic and sigmoid, different percentages of training to test and learning algorithms were used. To find the best training function all data used to build neural network. The R-squared of these training functions is presented in Fig. 9. Finally, the best solution in this algorithm can be obtained for the three-layer perceptron network, with the number of 20 neurons and the sigmoid activation function and the Levenberg–Marquardt learning algorithm. Also, about the support vector machine method, various kernel functions were investigated and the results are shwon in Fig. 10. Based on the results, the RBF kernel function provides the best response (Fig. 11).

**Figure 12:**
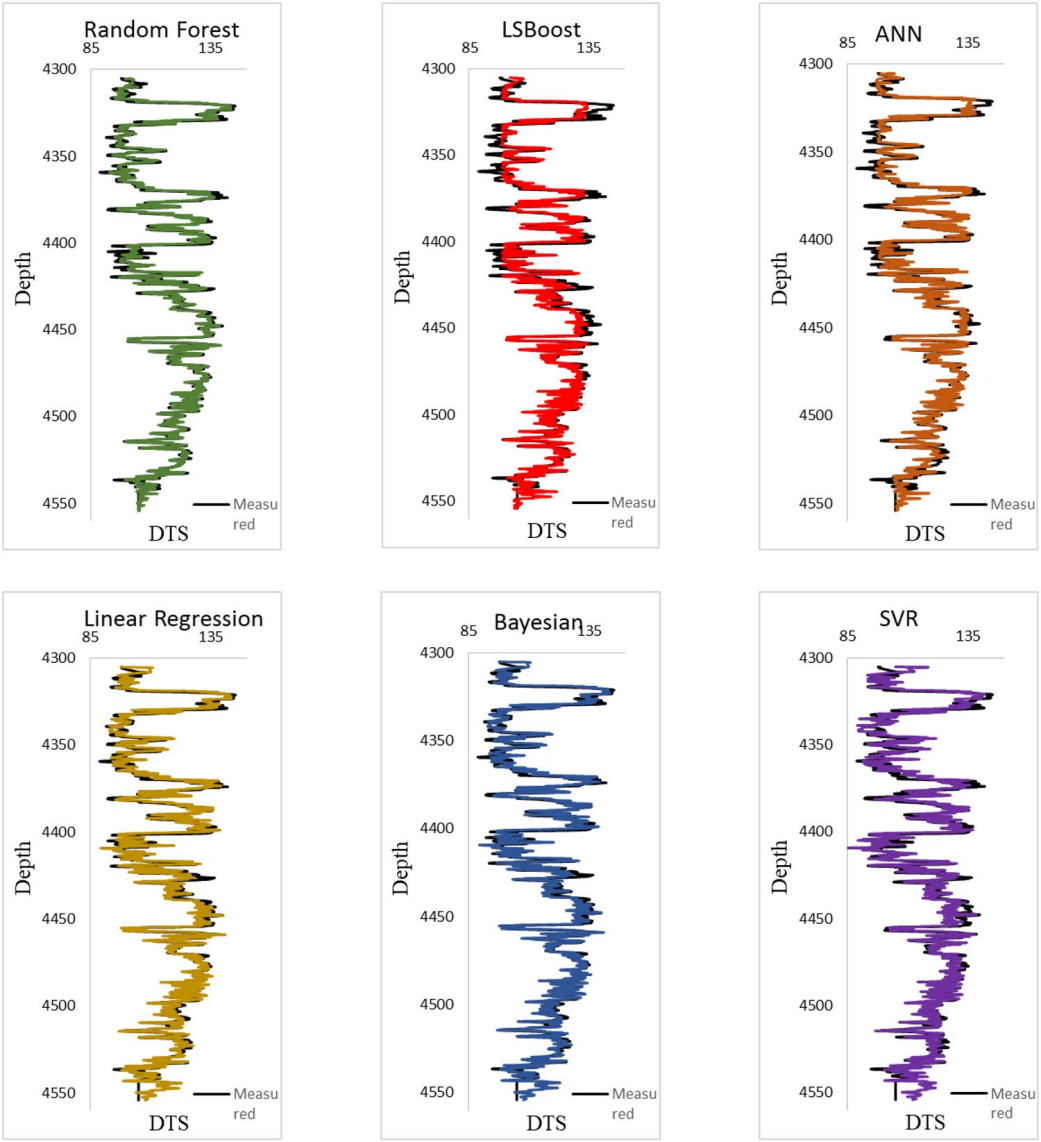
Visual comparison between performance graphs of each method.

Figure 12 shows a visual comparison between the performance graphs of each method. By using it, you can have a more accurate evaluation of the methods and observe the quality of each estimation. Based on R^2^ values and even by referring to the calculated errors in Table 3, ANN has a better performance compared to LSboost method. Meanwhile, based on Fig. 12, it can be seen that the LSboost method has performed better at depths greater than 4500 m. Therefore, a method may have overlay small error values, but it may not be effective in all depths. This doubles the importance of investigating the method results in different depths.

### Sensitivity analysis

Sensitivity analysis to input data is one of the important steps in the machine learning process. This analysis helps to understand the sensitivity of machine learning algorithms to changes in input data and also states whether their output changes under the influence of these changes or not. Sensitivity analysis to input data can help in better understanding the performance of machine learning algorithms and better selection of relevant parameters and settings. By using this analysis, it is possible to have a better understanding of the behavior of the algorithms against the changes in the input data and to create better models. To identify the impact and importance of each well logging parameter on the output of the model, the following equation is used to calculate the correlation coefficients^54^:

17 $$r\left({I}_{j},{DTS}_{j}\right)=\frac{\sum_{i=1}^{N}\left({ I}_{j,i}-\overline{{I }_{j}}\right)\left({ DTS}_{i}-\overline{{DTS }_{j}}\right)}{\sqrt{\sum_{i=1}^{N}{\left({ I}_{j,i}-\overline{{I }_{j}}\right)}^{2}\sum_{i=1}^{N}{\left({ DTS}_{i}-\overline{{DTS }_{j}}\right)}^{2}}}$$

where ${I}_{j,i}$ and $\overline{{I }_{j}}$ ® represents the i-th value of the j-th input parameter and its average, respectively. Similarly, ${DTS}_{i}$ and $\overline{{DTS }_{j}}$ represent the estimated shear wave velocity and its average, respectively. The dependence values of each method on the input parameters are shown in Table 2.

**Table 2:** The sensitivity of the response of each method to the input data.

	ANN	Bay	LB	LIN	RF	SVR
DTC	0.744	0.784	0.746	0.783	0.738	0.841
GR	− 0.391	− 0.448	− 0.402	− 0.447	− 0.422	− 0.507
Depth	0.198	0.202	0.228	0.199	0.205	0.232
HCAL	− 0.664	− 0.673	− 0.658	− 0.673	− 0.654	− 0.751
HTNP	0.946	0.971	0.971	0.971	0.944	0.903
RHOZ	− 0.774	− 0.795	− 0.788	− 0.796	− 0.768	− 0.719

**Figure 13:**
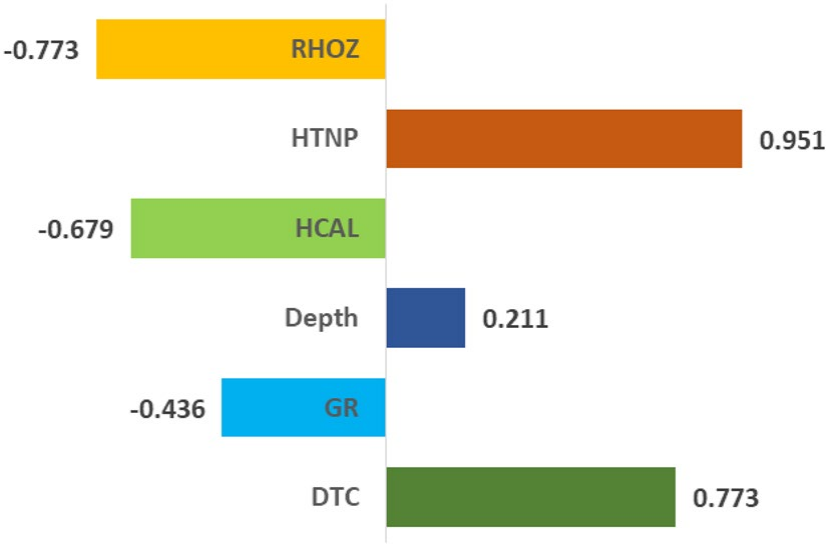
The sensitivity of the estimated DTS values to each of the input parameters.

As shown in Table 2, in general, the sensitivity of different parameters in each of the machine learning methods for DTS estimation has a trend and approximately a specific value. Based on this, parameters such as DTC, Depth and HTNP have a positive effect and GR, Hcal and RHOZ parameters have a negative effect on the final response of the models. This means, for example, increasing density will decrease DTS and increasing porosity will increase DCT. Also, among the input parameters, porosity, compressional wave transit time, and density respectively have the greatest impact on the final response. Figure 13 shows the average effect of each parameter in the DTS calculation.

### Evaluation of the performance of machine learning methods

Evaluating the performance of machine learning methods is of great importance so that the quality and performance of machine learning models can be investigated in a quantitative and evaluable way. Therefore, statistical parameters such as R^2^, ARE, RMSE, and MSE can be used. Several researchers have used these parameters to evaluate the performance of their estimation methods using machine learning.

- R-square:

The R-squared method or more completely R-squared coefficient of determination is a measure that is used in statistical analysis of error and performance evaluation of regression models. This approach quantifies the degree to which the model elucidates variations in the dependent variable (output) compared to changes in the independent variables (features).

The R-squared value ranges from 0 to 1 and is typically presented as a percentage. A value of 1 for R-squared signifies that the model has successfully accounted for all variations in the dependent variable using the independent variables, indicating high accuracy. If the R-squared is equal to 0, it indicates that the independent variables cannot predict or explain the dependent variable and the model is useless. If the R-squared is between 0 and 1, it indicates the ability of the model to explain changes in the dependent variable, and a higher value indicates a better agreement between the model and the data. This parameter can be calculated through the following relationship:

18 $${R}^{2}=1-\frac{\sum_{i=1}^{N}{\left({DTS}_{i}^{pred}-{DTS}_{i}^{exp}\right)}^{2}}{\sum_{i=1}^{N}{\left({DTS}_{i}^{pred}-average\left({DTS}_{i}^{exp}\right)\right)}^{2}}$$

- RMSE (root mean square error):

RMSE quantifies the error by computing the square root of the mean squared difference between the estimated values and the actual values. A larger RMSE indicates a larger difference between the estimated and actual values. This error measure is often used in regression problems. This parameter can be calculated from the following relationship:

19 $$RMSE\%=\frac{100}{N}{\sum }_{i=1}^{N}{\left(\frac{{\sum }_{i=1}^{N}{\left({DTS}_{i}^{pred}-{DTS}_{i}^{exp}\right)}^{2}}{N}\right)}^\frac{1}{2}$$

- ARE (absolute relative error):

ARE evaluates the error magnitude by determining the ratio of the absolute difference between the estimated values and the actual values to the actual value itself. This measure of error is proportional and shows how much the error is compared to the true value. It is often used in forecasting and estimating values in specific intervals. The relationship to calculate this parameter is as follows:

20 $$ARE\%=\frac{100}{N}{\sum }_{i=1}^{N}\left(\frac{{DTS}_{i}^{pred}-{DTS}_{i}^{exp}}{{DTS}_{i}^{exp}}\right)$$

- MSE (mean square error):

MSE quantifies the error magnitude by computing the mean squared difference between the estimated values and the actual values. Similar to RMSE, MSE is employed to assess the disparity between estimated and true values in regression problems. A higher MSE value indicates a larger discrepancy between the values.

21 $$MSE\%=\frac{1}{N}{\sum }_{i=1}^{N}{\left({DTS}_{i}^{pred}-{DTS}_{i}^{exp}\right)}^{2}$$

In the aforementioned interface, N represents the number of data points, ${DTS}_{i}^{pred}$ represents the estimated shear wave velocity, and ${DTS}_{i}^{exp}$ represents the actual shear wave velocity. Overall, these methods provide a measure of the error or disparity between the estimated values and the actual values within a model or method. Table 3 displays the calculated values of the statistical parameters for each of the methods. Based on the results, the random forest method with the highest R^2^ value and the lowest error values of RMSE, MSE, and ARE is considered the best method for DTS estimation.

**Table 3:** Accuracy of machine learning methods in DTS estimation based on statistical parameters.

	ANN	Bay	LIN	RF	LB	SVM
ARE	0.0436	0.1998	0.1998	0.1011	− 0.2063	0.6219
MSE	22.4068	28.0138	28.0240	9.4567	27.8158	37.5822
RMSE	4.7336	5.2928	5.2938	3.0751	5.2741	6.1304
R^2^	0.8780	0.8471	0.8470	0.9495	0.8583	0.7975

## Conclusion

This review encompassed a study focused on estimating shear wave transition time utilizing machine learning algorithms and well logging data from a carbonate reservoir in southwestern Iran. The findings of this study identified the random forest method as the most suitable approach for estimating shear wave velocity. Several machine learning algorithms, including perceptron multilayer neural networks, Bayesian, Generalized least squares, multivariate linear regression, and support vector machine, were examined during the study. However, none of these methods exhibited superior performance compared to the random forest approach. The evaluation of each method's performance was conducted using statistical parameters such as R^2^, ARE, RMSE, and MSE. This study also examined the influence of various petrophysical parameters on shear wave velocity estimation within each method. The findings indicated that the compression wave transit time and density had the most significant impact on the final response. A notable aspect of this study is the comparison of commonly employed machine learning methods for estimating shear wave velocity in carbonate reservoirs located in southwestern Iran. Based on the results obtained, the random forest method emerges as a recommended and reliable approach for accurately estimating shear wave velocity in such reservoirs.

## Data Availability

Data will be available upon request. Ali Ranjbar (Corresponding Author) will be contacted if someone wants to request the data from this study.

## Abbreviations

ANN: Artificial neural network
DTC: Compressional wave transit time
DTS: Shear wave transit time
GR: Gamma-ray log
HCAL: Caliper log
HTNP: Neutron porosity
LSBoost: Least-squares boosting
RF: Random forest
RHOZ: Density
SVR: Support vector regression
Vp: Compressional wave velocity
Vs: Shear wave velocity
